# Utilization of CO_2_ in supercritical conditions for the synthesis of cyclic poly (N-isopropylacrylamide) via emulsion and homogeneous reactions

**DOI:** 10.1038/s41598-022-19951-6

**Published:** 2022-10-19

**Authors:** Sahar Daneshyan, Gholamhossein Sodeifian

**Affiliations:** 1 Department of Chemical Engineering, Faculty of Engineering, University of Kashan, Kashan, 87317-53153 Iran; 2 Laboratory of Supercritical Fluids and Nanotechnology, University of Kashan, Kashan, 87317-53153 Iran

**Keywords:** Chemical engineering, Chemical engineering

## Abstract

In this study, cyclic poly (N-isopropylacrylamide) (cPNIPAAM) was synthesized in supercritical carbon dioxide (SC-CO_2_) using emulsion and homogeneous reactions for the first time. This was accomplished by applying free radical polymerization and nitroxide compounds to produce low molecular weight precursors in the SC-CO_2_ solvent. The cyclization reaction occurred in a homogeneous phase in the SC-CO_2_ solvent, with dimethylformamide (DMF) serving as a co-solvent for dissolving the linear precursor. This reaction was also conducted in emulsion of SC-CO_2_ in water. The effects of pressure and time on the morphology, molecular weight, and yield of a difunctionalized chain were investigated, where a higher pressure led to a higher yield. The maximum yield was 64% at 23 MPa, and the chain molecular weight (M_w_) was 4368 (gr/mol). Additionally, a lower pressure reduced the solubility of materials (particularly terminator) in SC-CO_2_ and resulted in a chain with a higher molecular weight 9326 (gr/mol), leading to a lower conversion. Furthermore, the effect of cyclization reaction types on the properties of cyclic polymers was investigated. In cyclic reactions, the addition of DMF as a co-solvent resulted in the formation of a polymer with a high viscosity average molecular weight (M_v_) and a high degree of cyclization (100%), whereas the CO_2_/water emulsion resulted in the formation of a polymer with a lower M_v_ and increased porosity. Polymers were characterized by ^1^HNMR, FTIR, DSC, TLC, GPC, and viscometry tests. The results were presented and thoroughly discussed.

## Introduction

Synthetic polymers are intriguing drug delivery candidates. Polymers usually show longer circulation time and the potential for tissue targeting^1^. The physical and chemical properties of the polymeric carriers are critical to the therapy’s success. Amongst these attributes, the shape of the carrier has been identified as one of the key factors^2,3^.

Cyclic polymers may be an innovative choice for this purpose due to their unique properties and cyclic structure. They take the form of rings or macrocycles, and due to the absence of chain ends, all repeating units are physically and chemically equivalent. In fact, the cyclic form excludes any possible reactions with terminal groups; thus, their properties are unaffected by such groups. They exhibit increased blood circulation times, increased permeability, and an effect on tumor tissue retention. In comparison to the linear type, they exhibit lower viscosity, higher glass transition temperature, higher critical solution temperature, smaller hydrodynamic volume and radius of gyration, higher rate of crystallization, higher refractive index, lower translational friction coefficient, and a more rapid decrease in the second virial coefficient with molecular weight^4–15^. Additionally, the delayed mass loss of biodegradable cyclic polymers prolongs their circulation in the bloodstream^4,16^.

Among cyclic polymers, cyclic poly (N-isopropylacrylamide) (cPNIPAAM) is a water-soluble polymer with a distinct thermal phase transition behavior, making it an intriguing candidate for use as a suitable carrier in drug delivery. This polymer has been synthesized by applying different methods. These include the atom transfer radical polymerization (ATRP) of functionalized chains followed by click cyclization^9,15^. Another method combines a click reaction based on anthracene and thiol with reversible addition-fragmentation chain transfer polymerization (RAFT). Moreover, it is formed by the ring closure of a heterodifunctional telechelic PNIPAAM precursor^15,17^.

Due to the absence of organic solvents and thus consideration of human and environmental protection, the use of supercritical carbon dioxide (SC-CO_2_) as a solvent in polymerization is an attractive alternative, particularly when producing medicine carrier polymers. This medium has been used commercially for an extended period in the pharmaceutical, textile, and food industries^18–20^. Because it is inexpensive, non-toxic, non-flammable, relatively inert, odorless, and requires no energy to remove, it is promoted as a green solvent. Furthermore, its properties are tunable via pressure and temperature adjustments^20,21^. Furthermore, it has many applications in various fields such as essential and seed oils extraction^22–27^, solubility^28–58^, nanoparticle formation^44,59–65^, impregnation^66,67^ and etc.

SC-CO_2_ is also a suitable solvent for nonpolar molecules with low molecular weight and small polar molecules. Moreover, water and ionic compounds are insoluble in SC-CO_2_. The mass transfer coefficients of components are greater in SC-CO_2_ than in other media. As a result of this property, the reaction rates in SC-CO_2_ improve^68^. Low viscosity, high mass transfer, and diffusivity contribute to reducing the probability of the Trommsdorf effect during polymerization. Other advantages of SC-CO_2_ include obtaining dry polymers after depressurizing the reactor and modifying the crystallinity of products.

Furthermore, polymers’ glass transition temperature (T_g_) is reduced in this solvent^69–71^ Therefore, SC-CO_2_ offers an excellent opportunity to process new advanced materials^21,72^. However, due to synthetic challenges, the applications of cyclic polymers remain unexplored^4^. As a result, the polymerization of functionalized precursors and the cyclic reactions that occur in SC-CO_2_ is also obscure. Following a review of the literature, it was observed that Mase et al. produced cPLA in the SC-CO_2_ for the first time in 2018^73^. Daneshyan and Sodeifian recently produced cyclic polystyrene in SC-CO_2_ and considered the different condition of reaction^74^.

According to the benefits of SC-CO_2_ media and since the synthesis of cPNIPAAM in SC-CO_2_ has not yet been reported; thus, in this research, cPNIPAAM was synthesized using the Deffieux method by applying unimolecular cyclization of linear $\alpha ,\omega$-heterodifunctional PNIPAAM as third polymer synthesized in SC-CO_2_^15^. To this end, radical polymerization via nitroxide compounds (4-hydroxy-TEMPO as the terminator) was utilized to create a difuntionalized chain of PNIPAAM in SC-CO_2_ solvent in the presence of 4, 4’ azobis (4-cyanovaleric acid) as the initiator. In this process, precipitation polymerization of NIPAAM was carried in SC-CO_2_. The initiator, terminator, and monomer are soluble in the SC-CO_2_ depending on the solvent's pressure and density^75–77^. The radicals are inert in the CO_2_, so there is no chain transfer to this solvent^76^. Difunctionalized chains started to precipitate when their molecular weight became higher than the critical molecular weight^75,78^. Functionalized polymer chains separated and gathered in the bottom of the reactor in the form of puffy ivory powder.

The cyclization reaction was performed in SC-CO_2_ using end-to-end equilibrium and the coupling agent 2-Chloro-1-methylpyridinium iodide. The reaction took place in two distinct reaction procedures. Dimethylformamide (DMF) was used as a co-solvent in the first process. In this condition, the coupling agent, activator and solution of polymer and co-solvent made a homogenous phase, according to the equilibrium phase of DMF and SC-CO_2_ and solubility of materials in SC-CO_2_^79,80^. As the time of reaction became over, the produced solution of cPNIPAAM in the form of yellow liquid was obtained.

The second reaction was performed in a CO_2_/water emulsion. In this condition, the reaction occurred in continues matrix of water; because of SC-CO_2_ is immiscible in water, therefore it makes an emulsion of CO_2_/Water. At the end of reaction solution of cPNIPAAM in water formed a yellow liquid. The final products lyophilized to obtain a polymer in the form of powder.

The products were characterized through different analyses, including ^1^HNMR, IR, GPC, SEM, DSC, and viscometry. Moreover, the effect of pressure and time on the morphology and molecular weight of the polymer and the effect of emulsion and homogenous reactions on the polymer’s characterization and cyclization yield were investigated and reported.

## Experimental section

### Materials

N-isopropylacrylamide (Sigma Aldrich, 99%), Triethylamine (BDH, HPLC Grade, 99.5%), Dimethylforamide (DMF) (BDH, spectroscopy Grade, 99.5%), 4,4′-azobis(4-cyanovaleric acid), (Sigma Aldrich, ≥ 75%) as initiator, 4-hydroxy-TEMPO (Sigma Aldrich, 97%) as terminator, 2-chloro-1-methylpyridinium iodide (Sigma Aldrich, 97%) as coupling agent for cyclization, Methanol (Merck, Chromatography Grade, 99.8%), deionized water and CO_2_ gas (99.99%, Fadak company of Iran) were purchased and applied for this research.

### Supercritical carbon dioxide production and reaction apparatus

**Figure 1:**
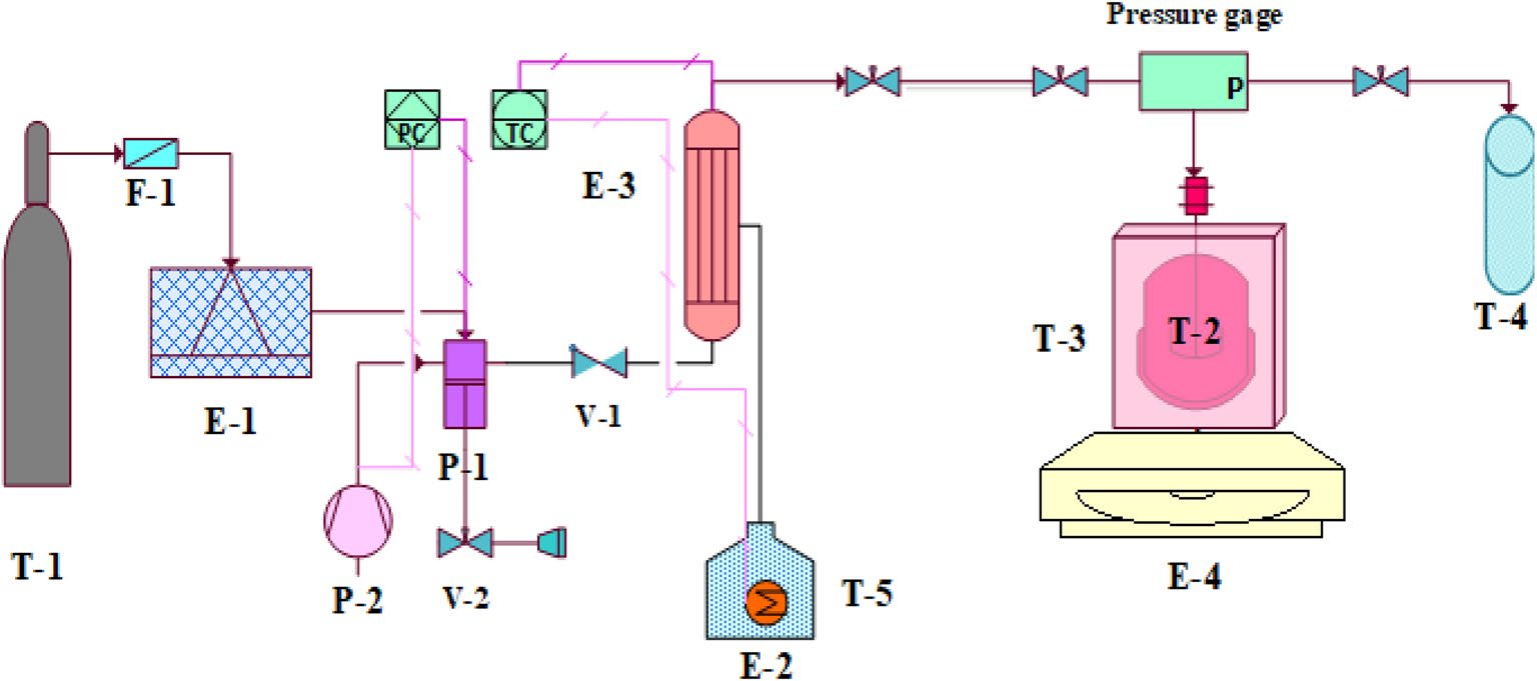
SC-CO_2_ producing and reaction apparatus. (E-1) Refrigerator, (E-2) water heater, (E-3) Heat exchanger, (E-4) Heater stirrer, (F-1) Filter, (P-1) High pressure pump, (P-2) Air compressor, (T-1) CO_2_ tank, (T-2) Polymerization reactor, (T-3) Paraffin bath, * (T-4) vent collector, (T-5) Water tank, (V-1) One-way pressure breaker valve, (V-2) Vent valve. *This is a glass open-door column.

As shown in Fig. 1, the experimental setup is included a refrigerator (E-1) to liquefy the gaseous CO_2_, and a high-pressure pump (P-1) to supply the required pressure. The pressurized liquid carbon dioxide was converted to the supercritical fluid by passing through a heat exchanger (E-3).

The high-pressure pump (P-1) and heat exchanger (E-3) were controlled by a control panel which adjusted the output pressure of the pump and output temperature of the fluid leaving the heat exchanger. The pressure of the pump was controlled by making off and on the air compressor (P-2). The exit temperature of the heat exchanger was controlled by switching on and off the heater located in the water supplying tank (E-2).

The polymerization and cyclization reactions occurred in a 150 ml cylindrical, stainless vessel (T-2). Considering the disc material and system limitations, the maximum temperature and pressure that could be supplied by reactor were (90 ± 1 °C) and (25 MPa ± 0.1), respectively. A magnetic stirrer (E-4) with maximum rotation velocity 1500 rpm was employed to improve the mixing of materials. The reactor temperature was maintained using a paraffin bath (T-3).

CO_2_ entered the filter (F-1), then passed through the line of the refrigerator (E-1). Cold CO_2_ was then pressurized by the high-pressure pump (P-1). To reach the supercritical point, the heat exchanger (E-3) enhanced the temperature of CO_2_ utilizing warm water. Finally, the SC-CO_2_ entered to the reactor (T-2). Paraffin bath (T-3) kept the reaction temperature constant. The required heat and agitation of materials were supplied by a heater-stirrer (E-4). Using this reactor, polymerization and cyclization reactions could be performed by SC-CO_2_ at different pressures.

### Methods

#### Producing linear difunctionalized precursor

The linear precursor was synthesized via controlled free-radical polymerization of NIPAAM with 4,4′azobis(4-cyanovaleric acid) as the initiator and 4-hydroxy-TEMPO as the terminator. The optimum *W*_*i*_*/W*_*m*_ and *W*_*t*_*/W*_*i*_ ratios were 1.5% and 83.3%, respectively. The exact amounts of materials are shown in Table 1.

**Table 1:** The amount of raw materials used for producing difunchtionalized polymer chains.

Component	Mol	Mass (gr)
NIPAAM	0.0176	2
Initiator	0.000107	0.03
Terminator	0.000145	0.025
SC-CO_2_	0.96 and 1.23^a^	42.24 and 54.12

^a^These quantities were used for two different pressures level.

The NIPAAM monomer, initiator, and terminator were added to the purged reactor to produce a linear precursor. SC-CO_2_ solvent was introduced into the reactor at various pressures following complete sealing. The stirring speed was 1500 rpm.

**Figure 2:**
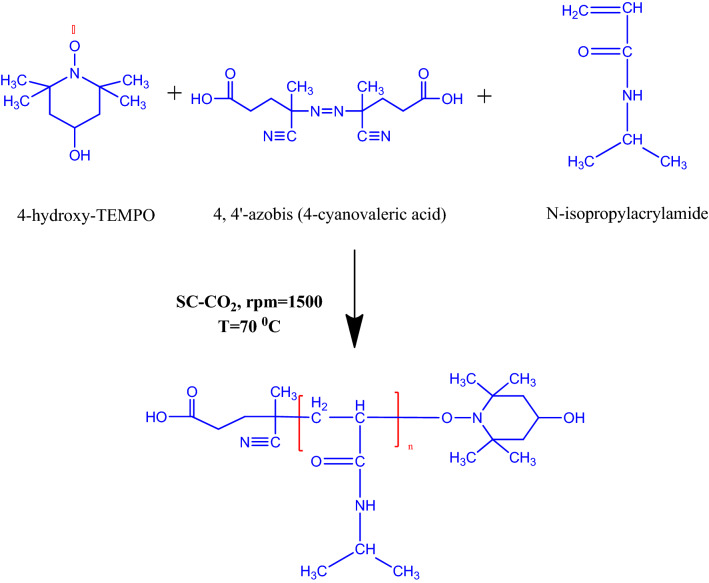
Synthesis of difunctionalized PNIPAAM in SC-CO_2_.

The initiator’s minimum activation temperature is 70 °C, in addition due to the thermosensitivity of the monomer and the previous researches, polymerization temperature was maintained at 70 °C^76,77,81,82^. According to previous researches, polymerization reaction below 10 MPa has no yield. Because the monomer is solid, it is insoluble in the SC-CO_2_ at these pressures^76,77^. Besides minimum pressure for dissolving terminator and initiator in SC-CO_2_ in temperature of 70 °C is 15 MPa^74^. Then by considering apparatus operation limitation, two pressure levels (20 and 23 MPa) were considered to study the effect of pressure. The reaction that occurred in the reactor is depicted in Fig. 2.

**Figure 3:**
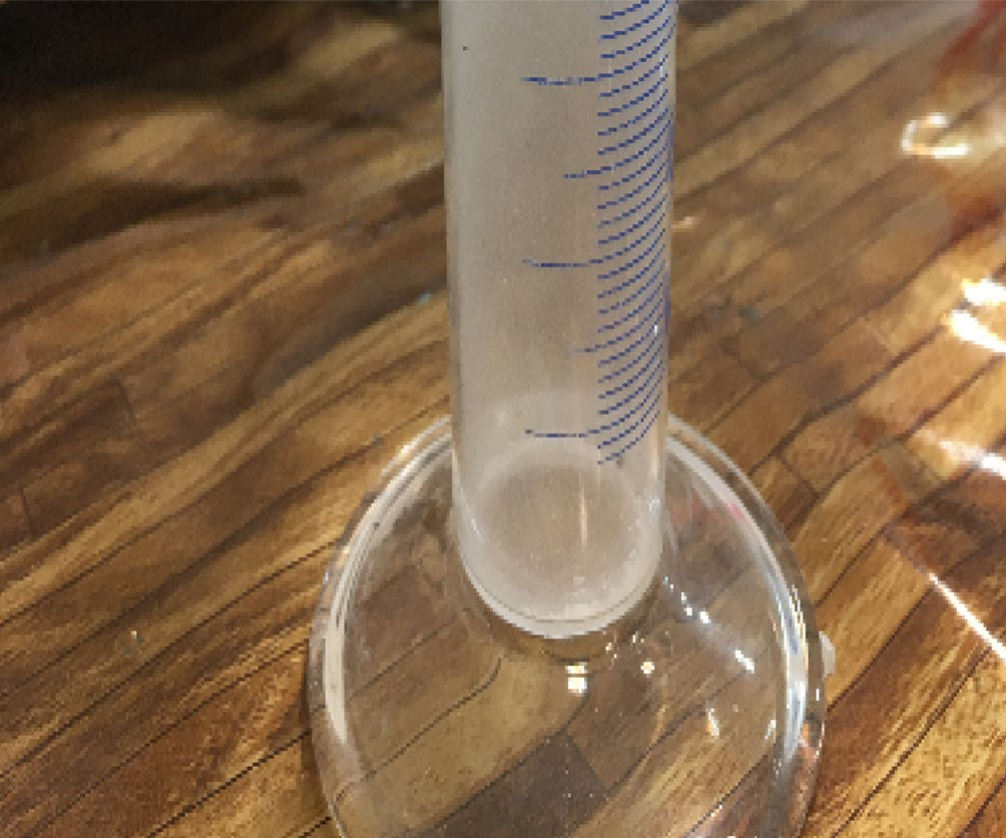
Residual raw materials, exited from the reactor by SC-CO_2_ after depressurizing.

The polymerization is classified as a heterogeneous polymerization. The monomer, initiator, and terminator were dissolved in SC-CO_2_ solvent^75–77^. In fact, SC-CO_2_ was performing as a solvent in this process. Since the viscosity of SC-CO_2_ is low (near the gas), which has a density near the liquid, then stirring at a high rate (1500 rpm), leads to a high mass transfer coefficient, and the high diffusivity of materials with high effective collision in the continuous phase resulted in a nearly homogeneous phase^21^. Difunctionalized polymer precipitated at the bottom of the reactor when their molecular weight became higher than critical molecular weight^75^. At the end of the reaction when the reactor was cooled down to the ambient temperature, the reactor was depressurized and discharged in one minute (by rate of 2.5 ml/s) by releasing the reactor's exit valve. Unreacted raw materials dissolved in SC-CO_2_ and were collected in the open-door vent collector, as shown in Fig. 3. This polymer is not soluble in SC-CO_2_, thus all the product remained at the bottom of the reaction vessel and did not exit by discharged solvent^79^.

**Figure 4:**
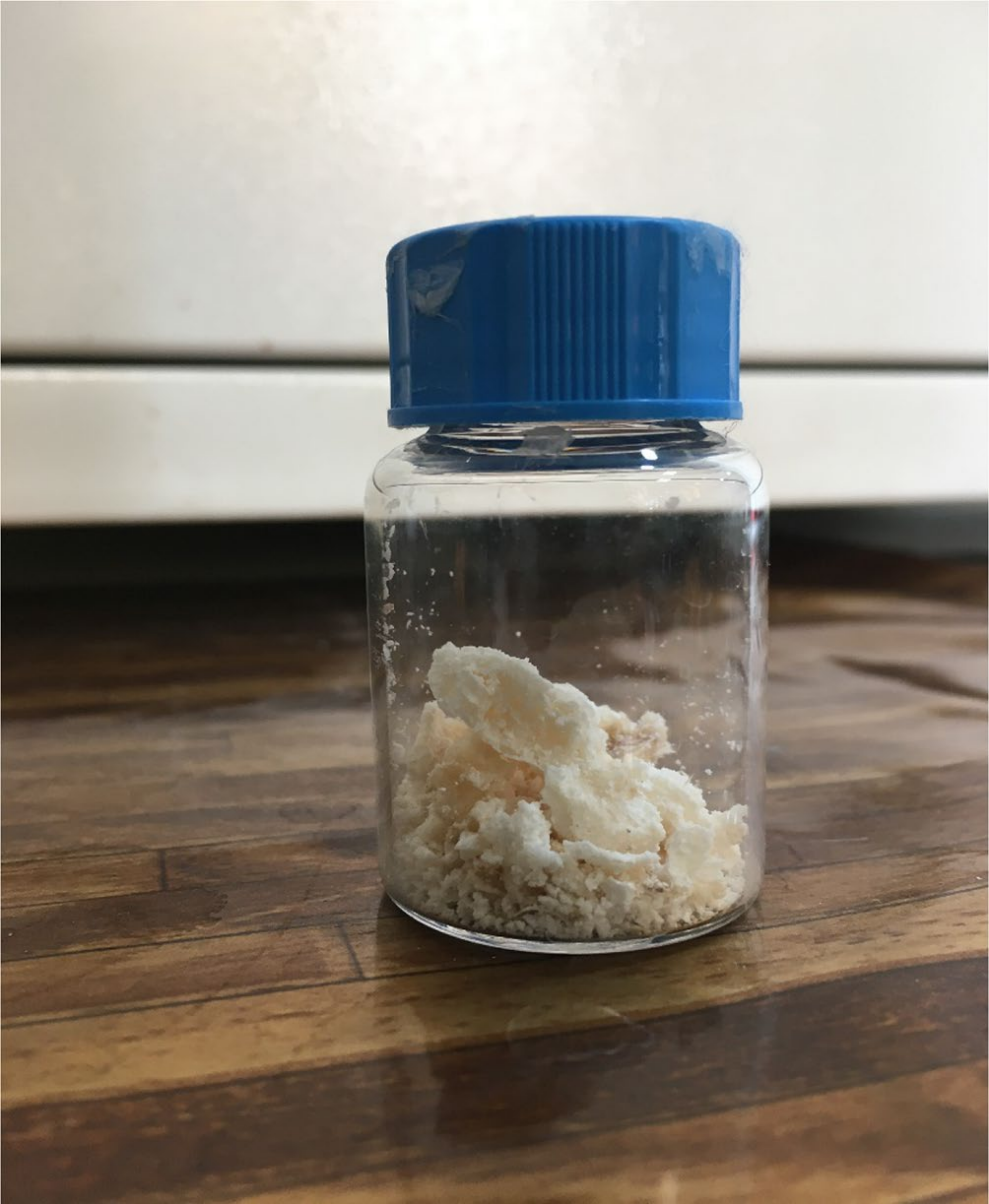
Difunctionalized PNIPAAM produced in SC-CO_2_.

As illustrated in Fig. 4, the difunctionalized precursor appeared as puffy ivory powder. The product weight ratio to the monomer weight was determined as the polymerization yield. The formation of the precursor was confirmed by characterization analysis. The effect of time and pressure of SC-CO_2_ was examined and discussed in the following sections.

#### Cyclization reaction and producing cyclic poly (N-isopropylacrylamide)

**Figure 5:**
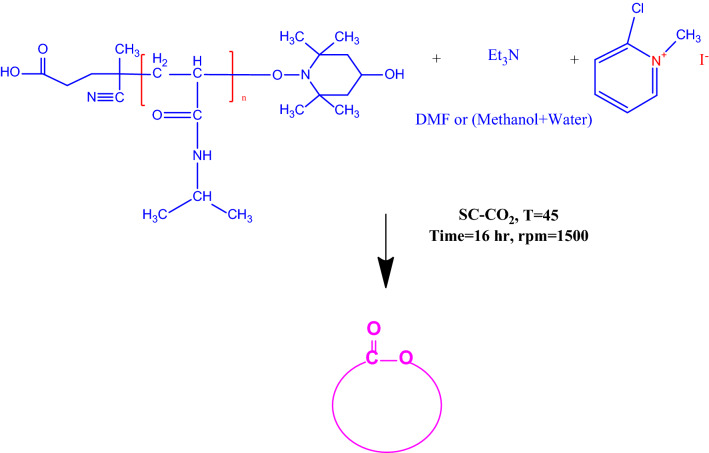
Synthesis of cPNIPAAM in SC-CO_2_ by using lactonization reaction.

Esterification or lactonization reactions were conducted by the end-to-end equilibrium method in this section, involving the reaction of alcoholic OH and carboxylic acid function and the separation of water molecules to produce a ketone or a large lactone ring^15,83^. As shown in Fig. 5, 2-Chloro-1-methylpyridinium iodide with a *W*_*pi*_*/W*_*p*_ = 1.5 and triethylamine with a *W*_*tea*_*/W*_*p*_ = 1.2 was used as coupling agent and activator, respectively. The exact amounts of materials are shown in Tables 2 and 3.

**Table 2:** Amount of raw materials used in the synthesis of cPNIPAAM in SC-CO_2_ and co-solvent of DMF.

Materials	Mol	Mass (gr)
Difunctionalized polymer^a^	0.000072	0.3
Coupling agent	0.00176	0.45
Activator	0.00356	0.36
SC-CO_2_	0.86	37.84
DMF	0.0387	2.832 (3 ml)

^a^Difunctionalized polymer was synthesized in condition of P = 23 MPa during 12 h, T = 70 $^\circ{\rm C}$, *W*_*i*_*/W*_*m*_ = 1.5%, *W*_*t*_*/W*_*i*_ = 83.3%, M_w_ = 4165 (gr/mol), M_n_ = 1488 (gr/mol) PDI = 2.8, and Mp = 1039 (gr/mol).

**Table 3:** Amount of raw materials used for the synthesis of cPNIPAAM in emulsion of CO_2_/Water.

Materials	Mol	Mass (gr)
Difunctionalized polymer^a^	0.000072	0.3
Coupling agent	0.00176	0.45
Activator	0.00356	0.36
SC-CO_2_	1.15	50.6
Methanol^b^	0.0742	2.376 (1 ml)
H_2_O	0.17	3 (3 ml)

^a^Difunctionalized polymer was synthesized in condition of P = 23 MPa during 12 h, T = 70 $^\circ{\rm C}$, *W*_*i*_*/W*_*m*_ = 1.5%, *W*_*t*_*/W*_*i*_ = 83.3%, M_w_ = 4165 (gr/mol), M_n_ = 1488 (gr/mol) PDI = 2.8, and Mp = 1039 (gr/mol).

^b^Methanol was used as co-solvent in water solvent for dissolving coupling agent.

##### Homogeneous cyclization reaction

**Figure 6:**
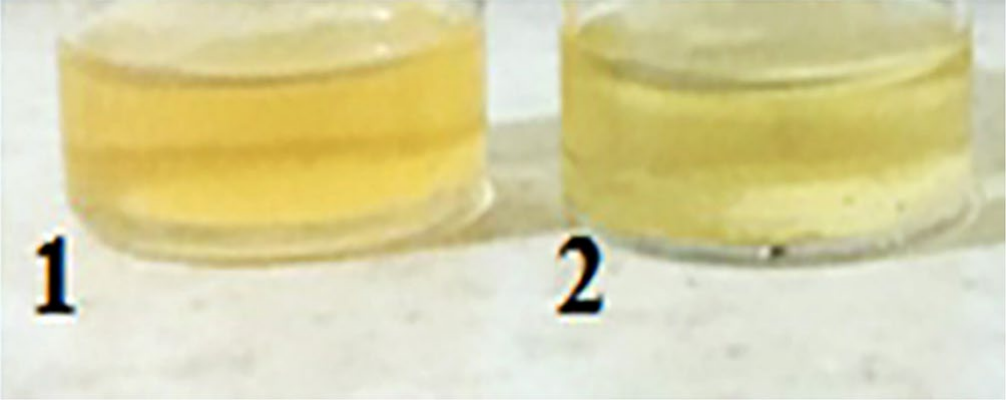
1—cPNIPAAM solution in DMF; 2—cPNIPAAM solution in water before lyophilizing.

**Figure 7:**
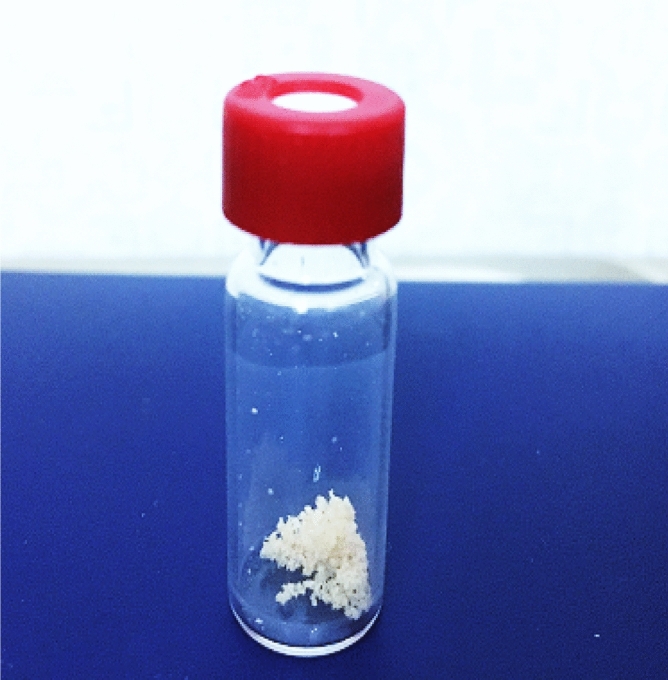
cPNIPAAM produced in homogenous phase of SC-CO_2_ solvent and DMF co-solvent after lyophilizing.

Due to the insoluble nature of the linear precursor in SC-CO_2_ solvent, a small amount of DMF was used as a co-solvent. The coupling agent and activator were added after the polymer was dissolved. The reactor was injected with SC-CO_2_ at a pressure of 15 MPa. This pressure, as shown in our recent research, provided the low molecular weight difunctionalized chains, completed cyclization yield^74^. Since the reaction was exothermic and reaction’s temperature was constant, then the pressure increased to 22 MPa. Thus, the average pressure which the reaction was performed in it was 18.5 MPa. The reaction was carried out for 16 h at a temperature of 45 °C. This condition was the same as the previous researches, for achieving high cyclization yield and thermosensitively of difuntionalized chains^74,84^. The stirring rate was set to 1500 rpm. Since DMF is partially soluble in SC-CO_2_, high-speed stirring, high diffusion, and a high mass transfer coefficient resulted in a nearly homogeneous phase according to the SC-CO_2_ and DMF phase equilibrium. cPNIPAAM and by-products were soluble in the co-solvent. After cool down and depressurizing SC-CO_2_ (during one minute (by rate of 2.5 ml/s)), some of by-products of the cyclization reaction and the co-solvent exited from the reactor; according to their solubility in the SC-CO_2_. The cyclic product remained at the bottom of the reaction cell in the form of a yellow liquid (see Fig. 6)^21,79,80^. The solution was lyophilized (freeze-drying). Figure 7 illustrates the final polymer in the form of ivory powder.

##### Emulsion cyclization reaction

As water is immiscible in SC-CO_2_, then its droplets dispersed in the water and formed an emulsion of SC-CO_2_ in water. This is a green method to produce porous hydrophilic polymers^21^. Cyclization reaction was performed in an aqueous phase. Finally, a porous polymer was formed.

For this purpose, the difunctionalized polymer was first dissolved in water prior to this reaction. In a droplet of methanol, the coupling agent and activator were dissolved. Following that, these two solutions were homogenized and added to the reactor. SC-CO_2_ was introduced at a pressure of 20 MPa after sealing due to make low size droplets and stable emulsion, according to apparatus’ operation limitation^85^. Since the reaction occurred in the water phase was endothermic, then in the constant temperature, it led to decrease the pressure of system about 7 MPa. Thus the average pressure which the reaction was performed in it was 16.5 MPa. The reaction was carried out for 16 h at a temperature of 45 °C for achieving high cyclization yield and thermosensitively of difuntionalized chains, respectively^74^.

**Figure 8:**
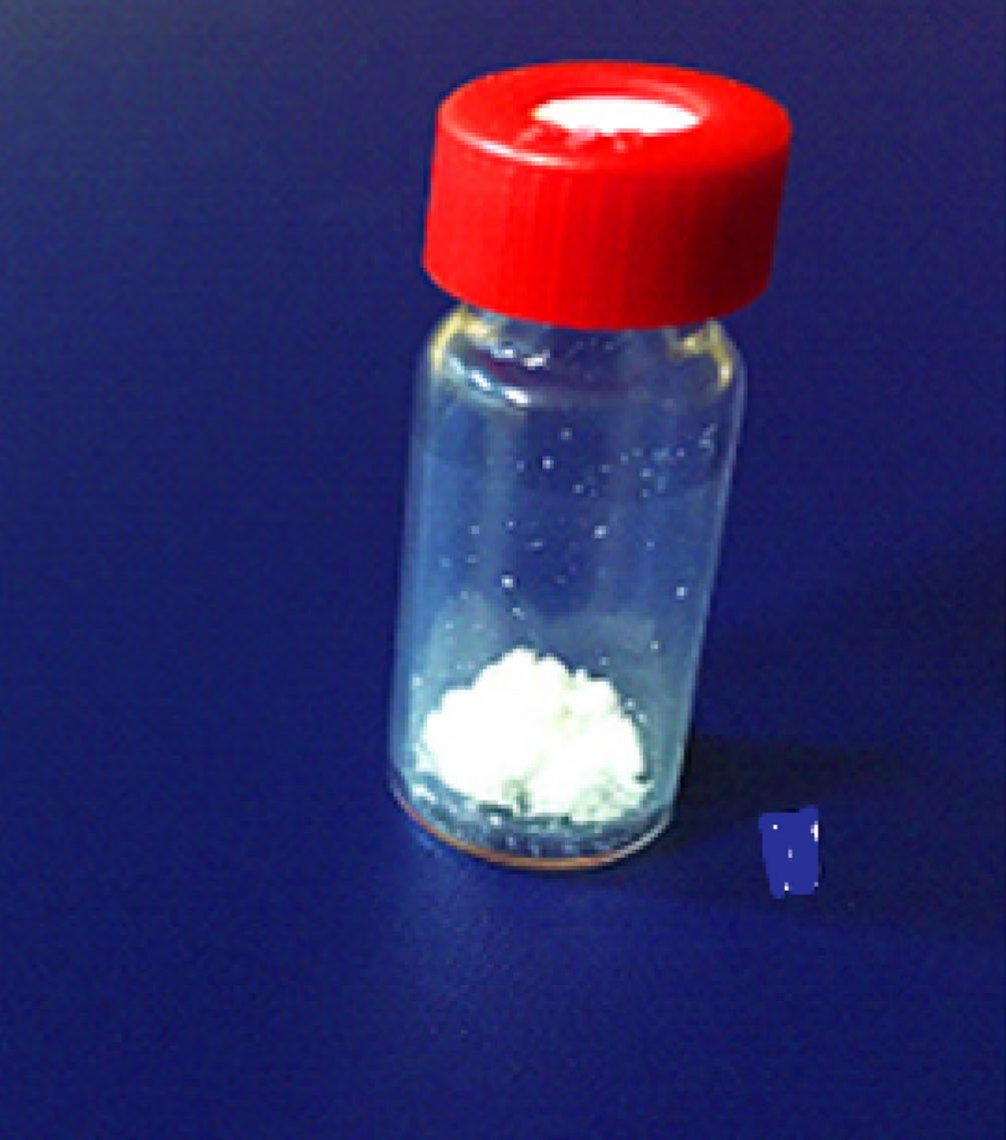
cPNIPAAM produced in emulsion of SC-CO_2_ in water after lyophilizing.

After cool down and depressurizing SC-CO_2_ (during one minute (by rate of 2.5 ml/s)), The yellow solution remained at the bottom of the reactor, as illustrated in Fig. 6. The solution was lyophilized, and the resulting polymer was a white powder, as depicted in Fig. 8. The products were identified, and the reaction type’s effect was evaluated and reported.

#### Hydrogen nuclear magnetic resonance

This test identifies the position of hydrogen atoms in the compound^85^. The analysis was accomplished by Bruker NMR spectrometer of Germany, with frequency about 400 MHZ, using dimethyl sulphoxide (DMSO) solvent.

#### Fourier transform infrared spectroscopy

Infrared spectra were recorded on FTIR Bruker-Alpha of Germany, which makes available the spectrums in the range of 400–4000 cm^−1^ using KBr tablet for a polymer powder.

#### Molecular weight distribution

M_w_ distribution was measured by gel permeation chromatography (GPC) (Agilent -1100 series, USA) using tetrahydrofuran (THF) solvent.

#### Measurement of the viscosity average molecular weight

The intrinsic viscosity of polymer solutions in water were measured at the concentration of 0.002 to 0.0033 (gr/ml) and ambient temperature, using Cannon–Fenske (Italy) viscometer 25 ml, with viscosity constant of 0.001477. The molecular weight of cPNIPAAM was determined, applying the Mark-Houwink equation ($\mu =k {[M]}^{a}$) by assuming its constants *a* = 1.1, for cyclic polymer produced by emulsion reaction and *a* = 1.5, for homogeneous reaction in co-solvent of DMF. The value of *k* = 0.46 × 10^–5^ (dl/gr) as previously reported for PNIPAAM solution in water^86,87^.

#### Differential scanning calorimetry

T_g_ and melting point (T_m_) of crystalline polymers were measured utilizing differential scanning calorimetry (DSC)^88^. DSC (Mettler TC-11 of Switzerland) was carried out in the temperature range of 27–250 °C at a heating rate of 10 (°C/min), under nitrogen atmosphere.

#### Scanning electron microscopy

The morphology of synthesized polymers is recognized by SEM. The scanning electron microscope Phenom (model Prox, Netherlands) was used. To obtain high quality images all of the samples were coated with a gold thin layer.

#### Thin layer chromatography

This experiment is applied for the identification and separation of different spices in the compounds on the basis of polarity. In this research, silica gel paper was used as a stationary phase, and the solution of ethyl acetate and n-hexane with the ratio of 1:4 has been considered as a mobile phase. Using this method, cyclization yield was qualitatively signified.

## Results and discussion

### Characterization of difunctionalized poly (N-isopropylacrylamide)

#### Hydrogen nuclear magnetic resonance analysis

**Figure 9:**
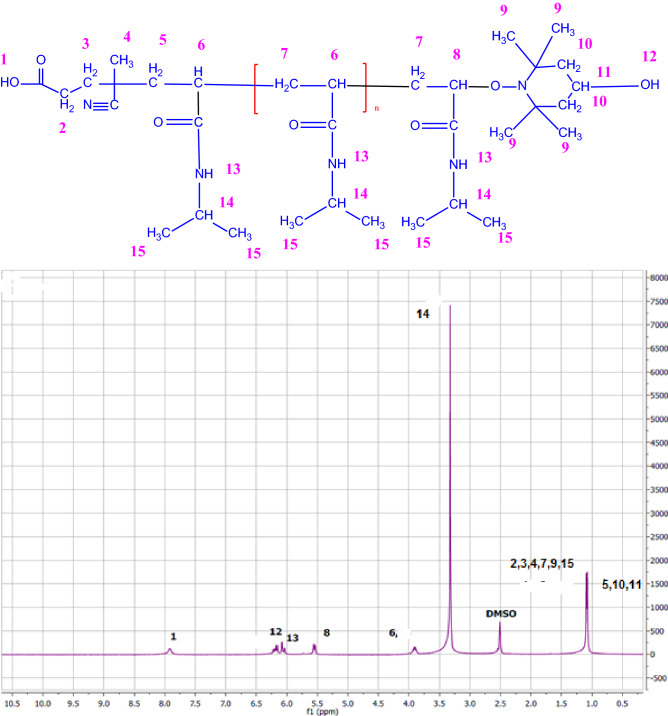
^1^HNMR diagram of difunctionalized PNIPAAM.

**Figure 10:**
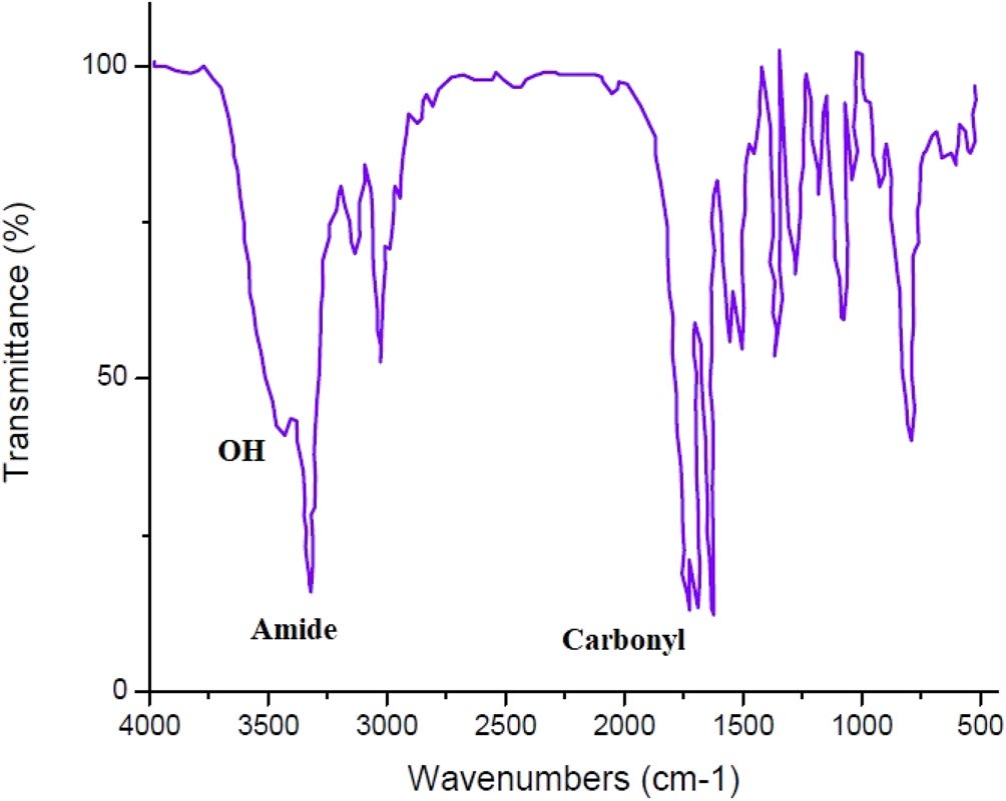
IR spectrum of PNIPAAM produced in SC-CO_2_.

Displacement at 8 and 6 ppm beside the specified functional groups and chemical bonds especially C-O bonding in IR spectrum (Fig. 10), defined the hydrogen of carboxylic acid and alcohol, respectively. Thus, polymerization by an initiator and attaching a terminator to a chain polymer’s end were justified. Then, due to these displacements, the desired product was revealed to be a difunctionalized chain. The details are included in Fig. 9^89–94^.

#### Fourier transform infrared spectroscopy spectrum analysis

As illustrated in Fig. 10, the peak at 3400 cm^−1^ confirmed the presence of the OH functional group, while the peak at 3300 cm^−1^ identified the amide functional group. Both of them created two valleys adjacent to one another. The carbonyl group appeared at 1700 cm^−1^. Carbonyl absorption adjacent to the OH group established the presence of carboxylic acid. Furthermore, the absorption at 1000 cm^−1^ confirmed the presence of C–O, indicating the presence of a radical attached to the hydroxy TEMPO compound. A weak absorption near 2000 cm^−1^ demonstrates the presence of nitrile in the initiator formula. Two absorption peaks at 1300 and 1500 cm^−1^ indicate the connection between N–O and the polymer chain^89^.

#### Molecular weight distribution

**Figure 11:**
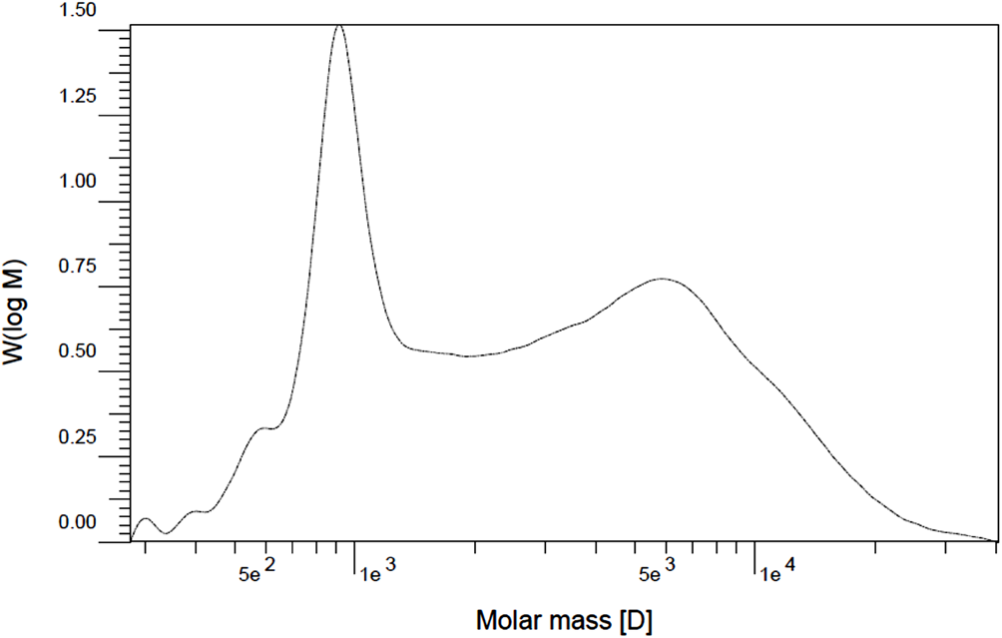
GPC chromatogram of PNIPAAM produced in SC-CO_2_ at P = 23 MPa during 6 h, T = 70 °C, *W*_*i*_*/W*_*m*_ = 1.5%, *W*_*t*_*/W*_*i*_ = 83.3%, M_w_ = 4368 (gr/mol), M_n_ = 1680 (gr/mol) PDI = 2.6, and Mp = 914.5 (gr/mol).

**Figure 12:**
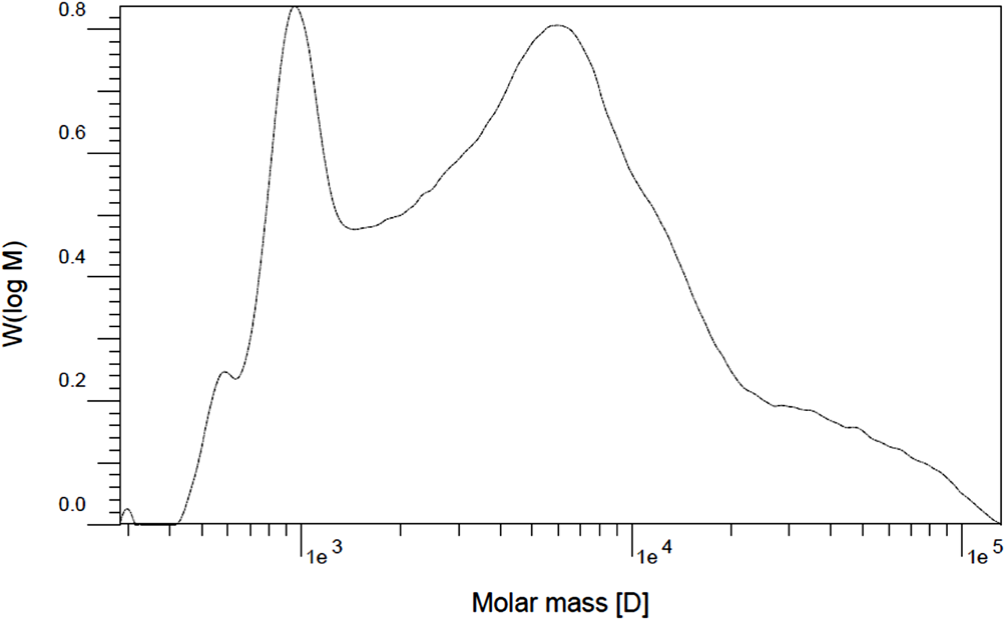
GPC chromatogram of PNIPAAM produced in SC-CO_2_ at P = 20 MPa during 6 h, T = 70 °C, *W*_*i*_*/W*_*m*_ = 1.5%, *W*_*t*_*/W*_*i*_ = 83.3%, M_w_ = 9326.5 (gr/mol), M_n_ = 2355 (gr/mol) PDI = 3.96, and Mp = 956.8 (gr/mol).

The molecular mass distribution chromatograms for PNIPAAM are shown in Figs. 11 and 12. Two summits appeared in the diagram under this synthesis condition, and the polydispersity index (PDI) values were broad. The first diagram depicts the polymerization at a pressure of 23 MPa, while the second depicts the polymerization at 20 MPa. For pressures of 23 MPa and 20 MPa, respectively, the PDI values are 2.6 and 3.96. Both PDI values are within the range of radical polymerization; the PDI value is dependent on the polymerization conditions, reaction mechanism, and subsequent environmental history of the polymer^94^. For polymerization in SC-CO_2_, pressure is an important parameter because of its effect on the density of the solvent. Increased pressure results in increased density and solubility of materials and a more homogeneous polymerization phase; as a result, the probability of chains with different weights existing is reduced, and the polydispersity becomes narrower^76^. In addition, higher solubility of terminator at the pressure of 23 MPa led to produce polymer chains with lower molecular weight.

### Effects of different variables of the linear precursor polymerization reaction

#### Effect of pressure

Two pressure levels (20 and 23 MPa) were considered to study the effect of pressure. According to previous researches, the polymerization reaction below 10 MPa has no yield. Because the monomer is solid, it is insoluble in the SC-CO_2_ at these pressures^76,77^. Then considering the yield, the optimum pressure was 23 MPa. As the most important effect of the pressure is manifested in the solvent density, changes in the pressure alter the liquid properties of the solvent, the solubility of components, effective collision among materials, agitation, and the life time of the radicals in the solvent is resulting in increased yield and a narrower polydispersity^21,75–77,95^. Another finding was the terminator’s solubility. Due to the low solvent density at lower pressures, the solubility of hydroxyl TEMPO was low; thus, effective collisions between chains and terminators reduced. As a result, the polymer’s M_w_ value increased. Table 4 contains further details in this regard.

**Table 4:** The effect of SC-CO_2_ pressure on the linear precursor synthesis T = 70 °C *W*_*i*_*/W*_*m*_ = 1.5%, *W*_*t*_*/W*_*i*_ = 83.3%, and time = 6 h.

Pressure (MPa)	Yield (% of converted monomer)	M_n_ (gr/mol)	M_w_ (gr/mol)^a^	Product morphology
20	36%	2355	9326	Glassy powder
23	64%	1680	4368	Glassy solid gel

^a^Molecular weight measured with GPC.

#### Effect of time

The effect of polymerization time was investigated between 6 and 16 h. Below 6 h the reaction did not have noticeable yield and the polymer was not in the form of powder, but it was in the form of light gel. Since this polymerization process is classified as active radical polymerization, the time parameter can significantly affect the yield. On the other hand, due to the reaction is endothermic, the pressure of SC-CO_2_ decreased (about 2 MPa) for an extended period (longer than 8 h), necessitating compensation. Pressure fluctuations decreased the solubility of materials, which resulted in a decrease in yield and M_w_ due to decrease in the monomer participated in to the reaction. Because of the longer radical lifetimes in SC-CO_2_ media^21^, the longer reaction time increased the probability of the presence of different molecular weight chains, resulting in the broad polydispersity. But the PDI values were within the radical polymerization range^96^. As shown in Table 5, the maximum monomer conversion yield was 64% for the time of 6 h.

**Table 5:** The effect of time on the linear precursor synthesis.

Time (h)	Pressure (MPa)	Yield (% of converted monomer )	M_n_ (gr/mol)	M_w_ (gr/mol)	PDI	Product morphology
6	23	64%	1680	4368	2.6	Glassy solid gel
12	23	58.5%	1488	4165	2.8	Glassy puffy powder
16	23	40%	*	*	*	Glassy powder

### Characterization of cyclic poly (N-isopropylacrylamide)

#### Fourier transform infrared spectroscopy spectrum analysis

**Figure 13:**
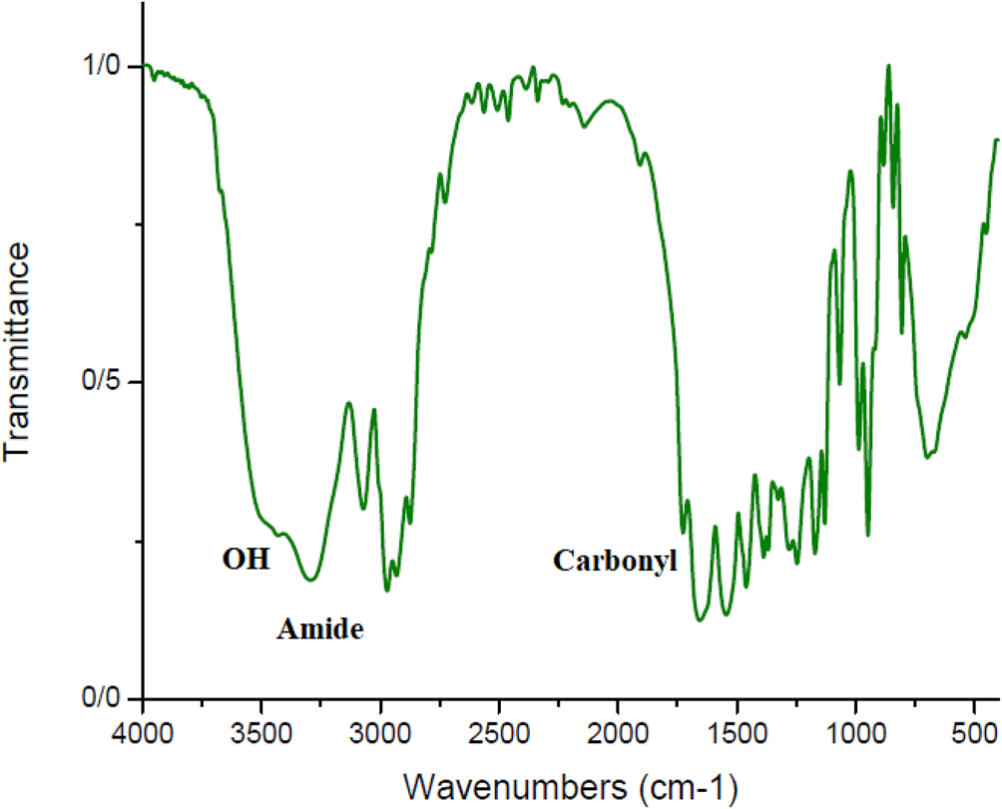
IR spectrum of cPNIPAAM produced in emulsion of SC-CO_2_ in water.

**Figure 14:**
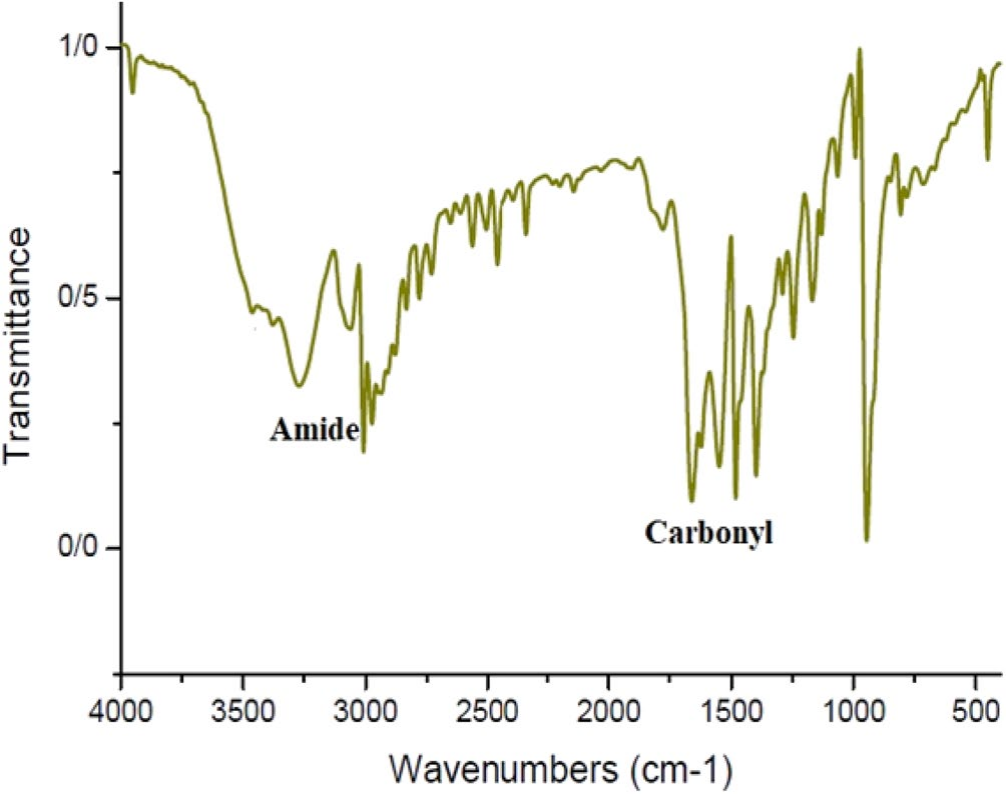
IR spectrum of cPNIPAAM produced in homogeneous phase of SC-CO_2_ and DMF co-solvent.

As illustrated in Figs. 13 and 14, for both cyclic polymers synthesized via distinct reactions, the OH (3400 cm^−1^) peak nearly vanished; additionally, the 3300 cm^−1^ peak associated with the amide group became shorter. The peak of C=O was slightly displaced. This evidence confirmed the formation of cyclic polymer and lactone ring^89^.

#### The viscosity average molecular weight measurement

M_v_ values for cPNIPAAM were approximately 2700 and 3400 gr/mol after lyophilization and solvent elimination via Mark-Houwink equation estimation for emulsion reaction and reaction in SC-CO_2_ solvent and DMF co-solvent, respectively. These results are acceptable when the linear chain’s mass (M_w_ = 4165 gr/mol), separated water and the relation between different measured molecular weights (M_n_ < M_v_ < M_w_) are considered^14^. Additionally, the intrinsic viscosity values indicated that the polymer produced in the emulsion condition had a lower viscosity average molecular weight than the polymer produced in the homogeneous phase of SC-CO_2_ and DMF co-solvent. This result was obtained from the porosity volume of the polymer generated during the emulsion reaction.

### Differential scanning calorimetry analysis

**Figure 15:**
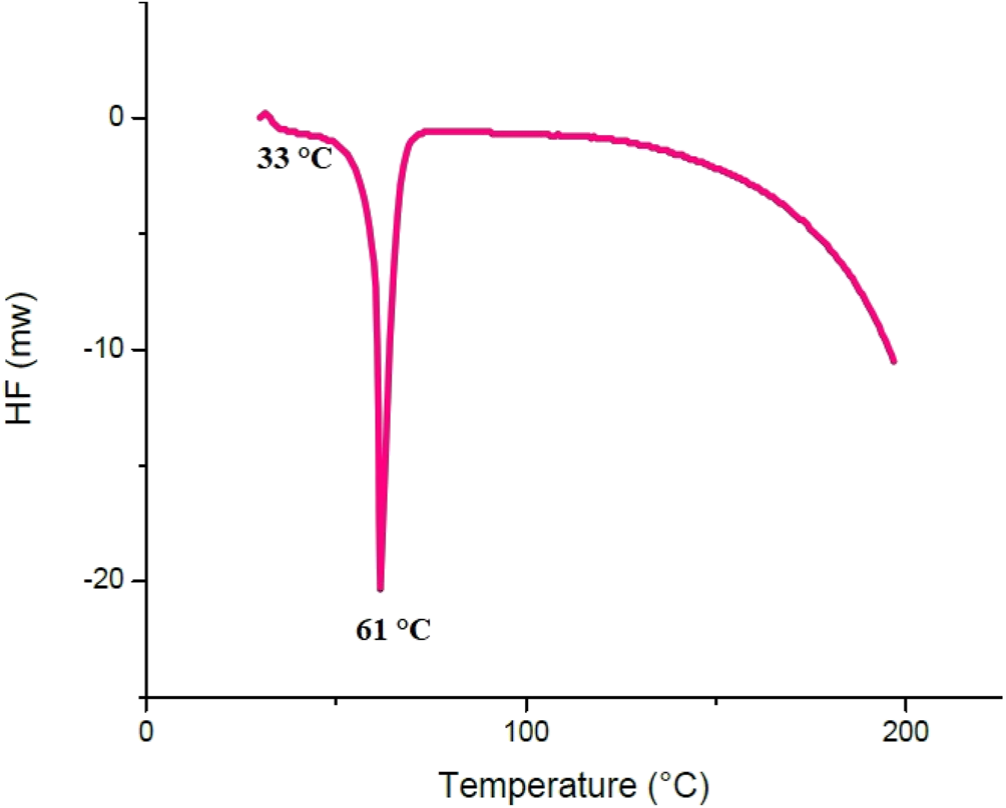
DSC diagram of difunctionalized PNIPAAM (M_w_ = 4165 gr/mol).

As illustrated in Fig. 15, the T_g_ of difunctionalized PNIPAAM (M_w_ = 4165 gr/mol) was 33 °C, with a melting point of 61 °C. The polymer begins to decompose at 140 °C. The DSC curve is identical to that of crystalline polymers.

**Figure 16:**
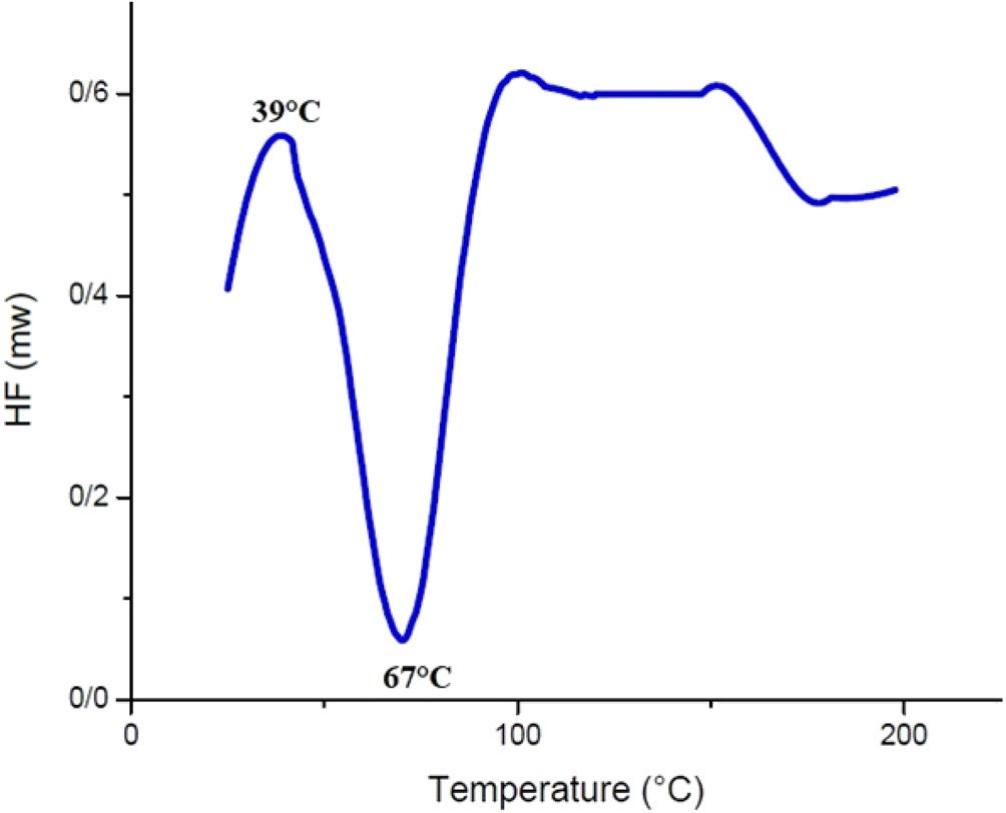
DSC diagram of cPNIPAAM synthesized by emulsion reaction.

**Figure 17:**
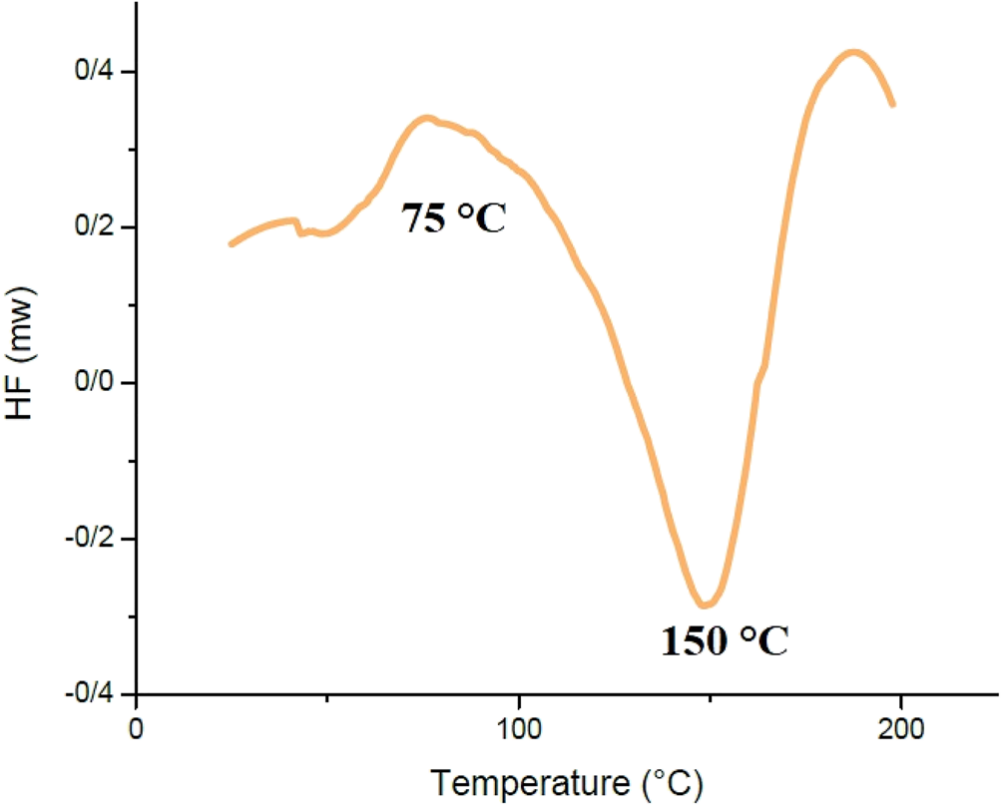
DSC diagram of cPNIPAAM produced in a homogeneous phase of SC-CO2 and co-solvent of DMF.

Figure 16 indicates that the T_g_ of cPNIPAAM synthesized via an emulsion reaction was approximately 39 °C and that it melted at 67 °C. These parameters were T_g_ = 75 °C and T_m_ = 150 °C for cPNIPAAM synthesized in the homogeneous phase of SC-CO_2_ and DMF (see Fig. 17). The DSC curves matched the crystalline structures.

### Thin layer chromatography results

**Figure 18:**
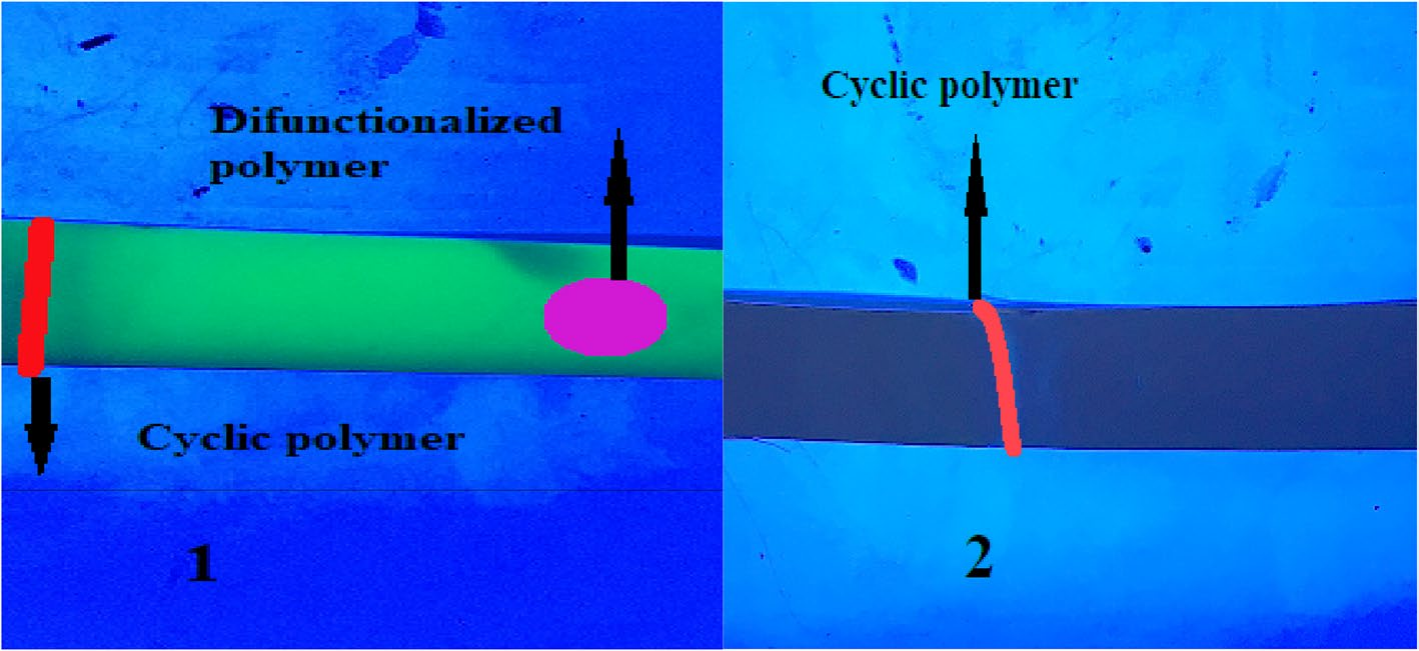
(**1**) TLC of cPNIPAAM produced in emulsion of SC-CO_2_ in water; (**2**) TLC of cPNIPAAM produced by cyclization in homogeneous phase of SC-CO_2_ and DMF.

Polar molecules separated more quickly here due to the polarity of the stationary phase. As illustrated in Fig. 18, for the cPNIPAAM produced in an emulsion of SC-CO_2_ and water, the cyclization of the difunctionalized precursor was not complete, and some of them remained. Cyclization in the homogeneous phase of SC-CO_2_ and DMF co-solvent converted all difunctionalized chains to lactone rings, increasing the cyclization yield to nearly 100%, because there is no amount of difunctionalized chains.

### Scanning electron microscopy image of polymers

**Figure 19:**
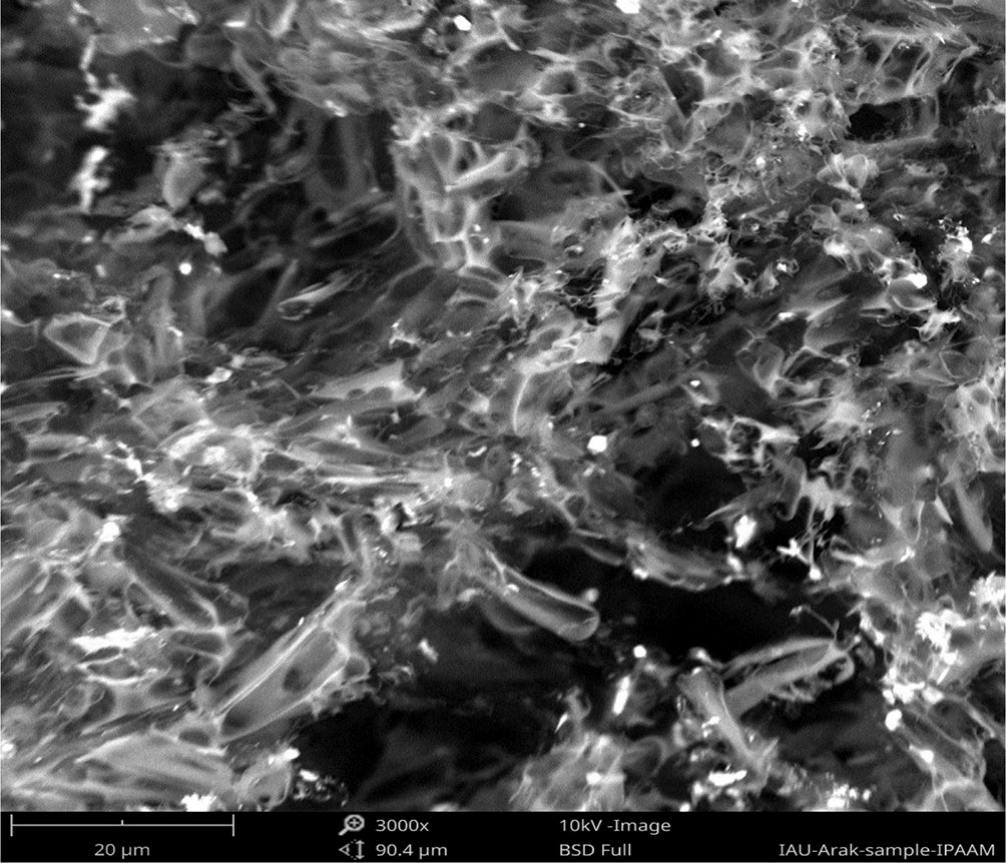
SEM image of difunctionalized PNIPAAM with magnification 3000.

**Figure 20:**
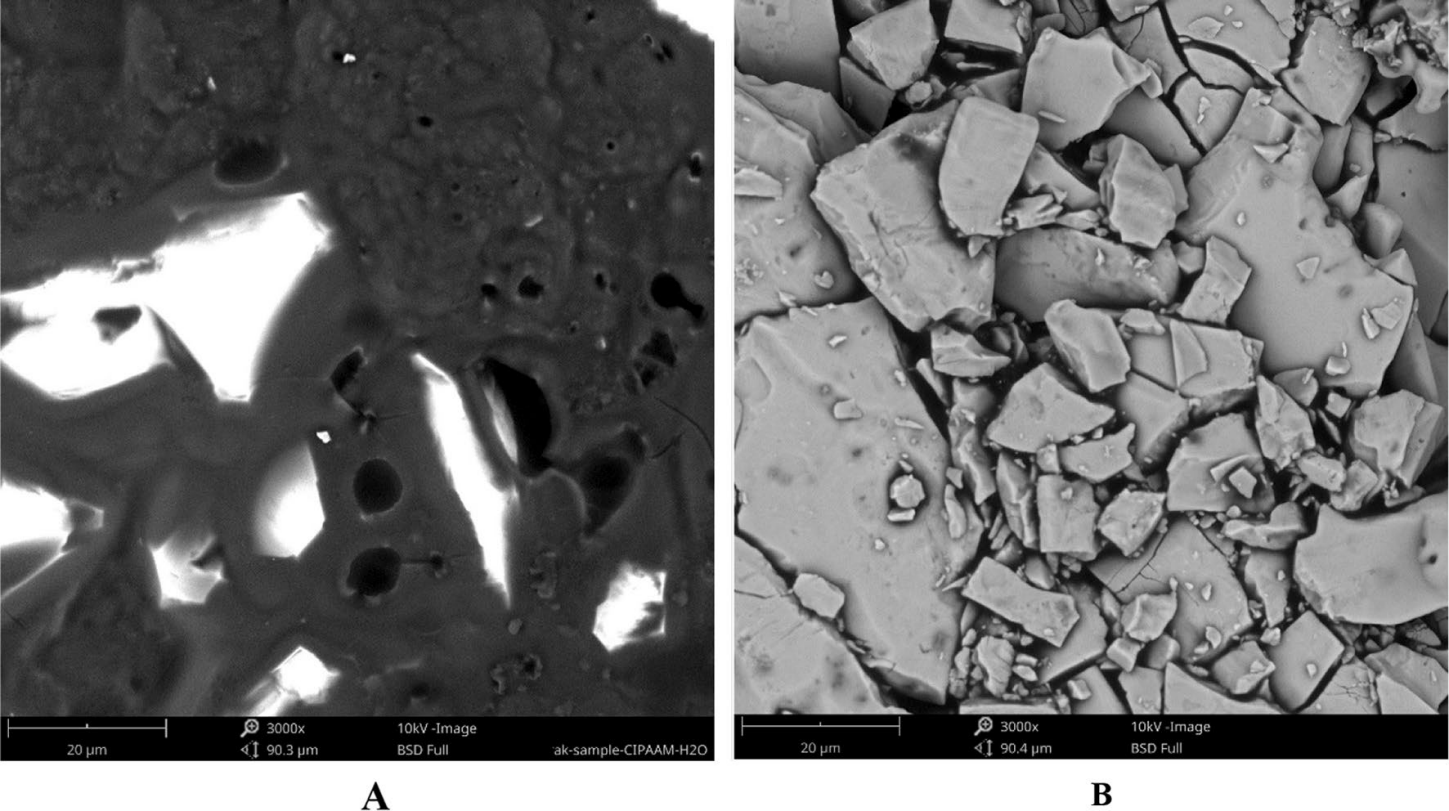
SEM image of cyclic polymers with magnification 3000. (**A**) cPNIPAAM produced in emulsion of SC-CO_2_ in water; (**B**) cPNIPAAM produced by cyclization in homogeneous phase of SC-CO_2_ and DMF.

As shown in Fig. 19, the difunctionalized polymer exhibited a puffy porous structure. The cyclic polymer formed regular blocks in the homogeneous phase of SC-CO_2_ and DMF co-solvent. The cPNIPAAM formed in an emulsion of SC-CO_2_ and water also had a regular structure but was less dense. Additionally, porosity was evident due to the emulsion reaction (Fig. 20). Thus, the homogeneous reaction produced a stronger cPNIPAAM than the emulsion reaction produced; consequently, it had higher thermal parameters and M_v_.

## Advantage of polymerization and cyclization in supercritical carbon dioxide

The organic solvent was omitted from the polymerization of NIPAAM to produce linear precursors. Then a pure polymer was obtained, resulting in a more environmentally friendly process and product. Besides the polymers produced by this solvent have the lower molecular weight and of course lower T_g_ and melting point in the form of crystalline polymers. Thus, these polymers are noticeable for biomedical purposes, especially drug delivery. As applying moderate pressure leads to reasonable yield in short time of reaction; it is another advantage of this method.

In addition, cyclization reaction by this solvent (SC-CO_2_) implemented to achieve cyclization yield about 100% at a moderated pressure of 18.5 MPa, and reduced consumption amount of organic solvent. Emulsion reaction was also carried out in water as clean and human safe solvent, increased the porosity of the polymer. Both cyclic polymers are in the form of regular blocks and classified in crystalline polymer that improved the specifications of them, resulting in interesting properties for drug delivery^97^.

## Conclusion

cPNIPAAM was synthesized in SC-CO_2_ via a homogeneous reaction with a co-solvent of DMF and in a SC-CO_2_ emulsion in water. Initially, $\alpha ,\omega$- difunctionalized precursor was synthesized via precipitation polymerization in SC-CO_2_ solvent. Solvent pressure and polymerization time were critical parameters in this process. Given to the nature of the reaction (endothermic), over a longer period, pressure fluctuation occurred, necessitating compensation; as a result, the yield and molecular weight decreased while the PDI broadened. Additionally, increased pressure increased the solubility of materials, particularly terminators, resulting in a decrease in molecular weight, a narrower polydispersity, and a higher yield. Cyclization in the homogeneous phase with SC-CO_2_ as solvent and DMF as co-solvent resulted in the formation of regular polymer blocks with increased M_v_. The cyclization yield was also 100% at 18.5 MPa pressure in the homogeneous phase. On the other hand, the emulsion reaction of SC-CO_2_ in water resulted in a prose polymer with a lower M_v_, T_g_, and T_m_.

## Data Availability

The datasets generated and/or analyzed during the current study are not publicly available due to confidential cases are available from the corresponding author on reasonable request.

## Abbreviations

ATRP: Atom transfer radical polymerization
cPNIPAAM: Cyclic poly (N-isopropylacrylamide)
DMF: Dimethylformamide
DMSO: Dimethyl sulphoxide
DSC: Differential scanning calorimetry
FTIR: Fourier transform infrared spectroscopy
GPC: Gel permeation chromatography
^1^HNMR: Hydrogen nuclear magnetic resonance
LCST: Lower critical solution temperature
M_n_: The number average molecular weight
M_v_: The viscosity average molecular weight
M_w_: Weight average molecular weight
NIPAAM: N-isopropylacrylamide
PNIPAAM: Poly (N-isopropylacrylamide)
PDI: Polydispersity index
RAFT: Reversible addition–fragmentation chain transfer
T_g_: Glass transition temperature
THF: Tetra hydro furan
TLC: Thin layer chromatography
T_m_: Melting point
W_i_: Weight of initiator
W_m_: Weight of monomer
W_p_: Weigh of linear polymer
W_pi_: Weight of 2-chloro-1-methylpyridinium iodide
W_t_: Weight of terminator
W_tea_: Weight of triethylamine
